# Supplementary material for: Relationship between Excreted Uremic Toxins and Degree of Disorder of Children with ASD
Source: Int J Mol Sci. 2023 Apr 11;24(8):7078. doi: 10.3390/ijms24087078 (PMC10138607; doi:10.3390/ijms24087078)
Supplement: Supplementary file 1 [file ijms-24-07078-s001.zip › ijms-2305210-supplementary.pdf]

Supplementary Materials

Investigating the effect of sex, age on the levels of urinary concentrations of determined uremic toxins

(1) Effect of gender in a group of children with ASD.

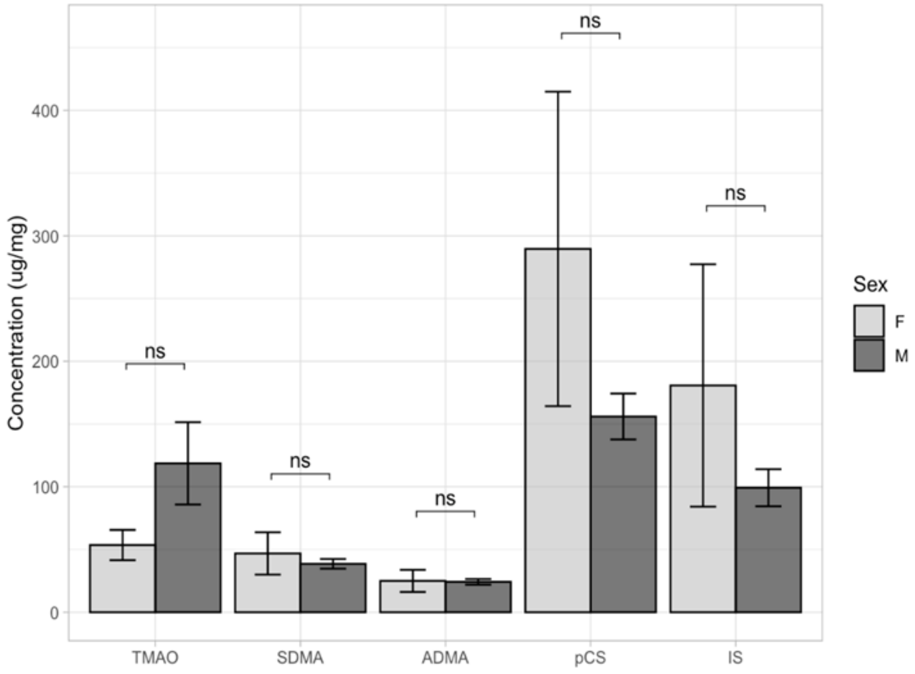

Figure S1. Levels of uremic toxins in girls and boys in ASD group.

(2) Effect of age in a group of children with ASD.

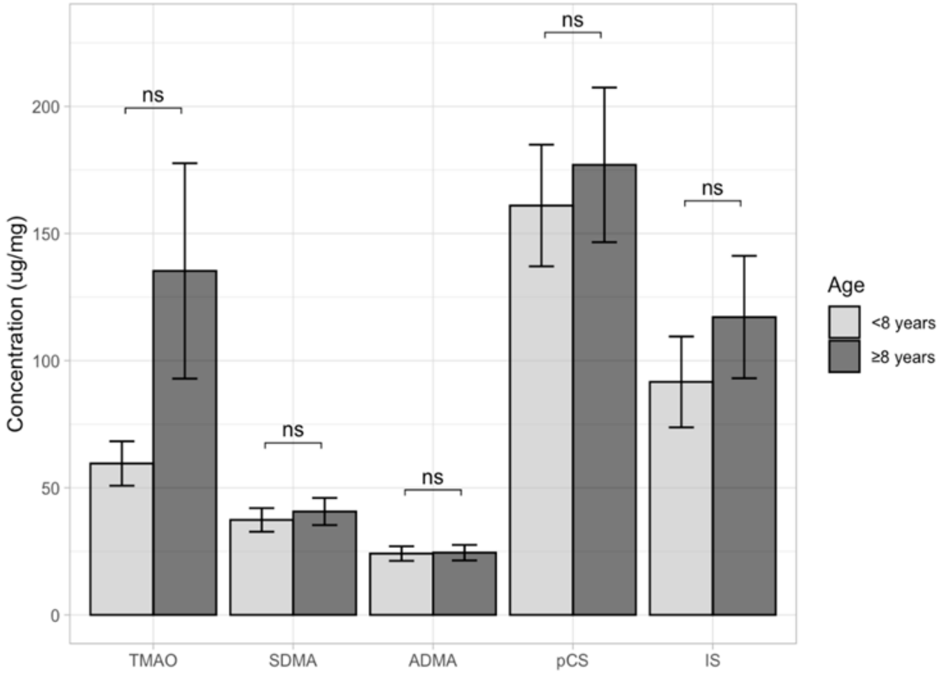

Figure S2. Levels of uremic toxins in girls and boys in ASD group depending of the age.

(3) Comparisons of TMAO, SDMA, ADMA, pCS, and IS values between ASD patients and control group.

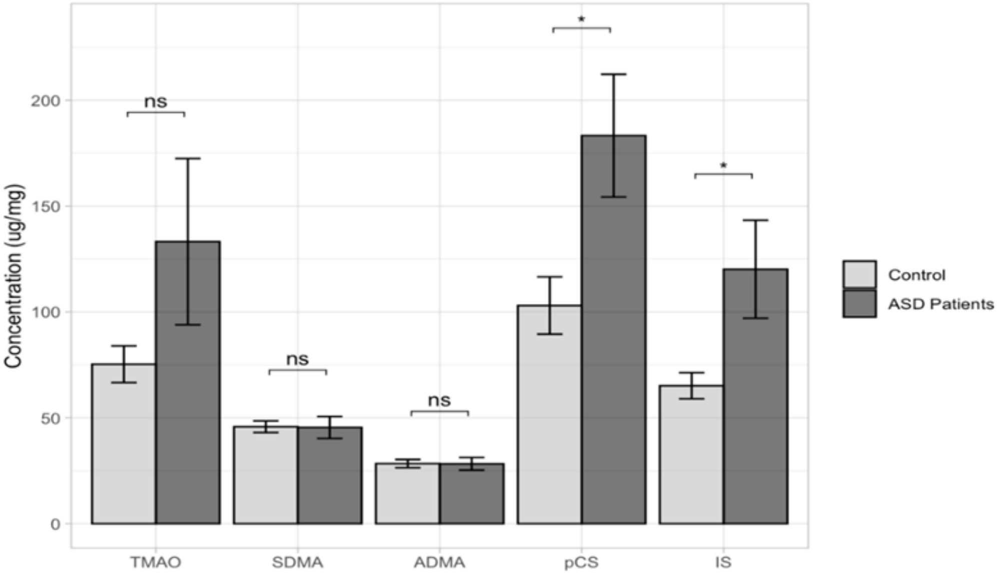

55

Figure S3. Levels of uremic toxins in the group of patients with an ASD diagnosis only compared to those in the control group.

(4) Comparison of TMAO, SDMA, ADMA, pCS, and IS values between ASD additional diagnosis group and control group.

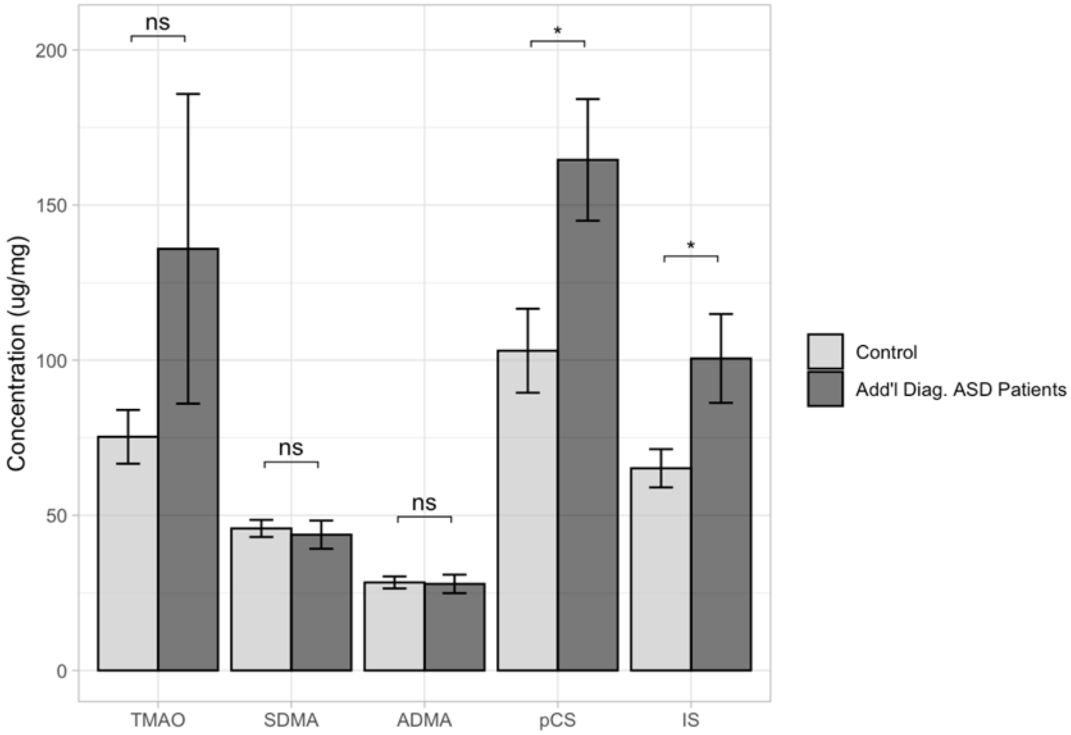

Figure S4. Levels of uremic toxins in the ASD additional diagnosis group compared to those in the control group.

(5) Comparison of TMAO, SDMA, ADMA, pCS, and IS values between HFA patients and controls, HFA patients with additional diagnosis and controls.

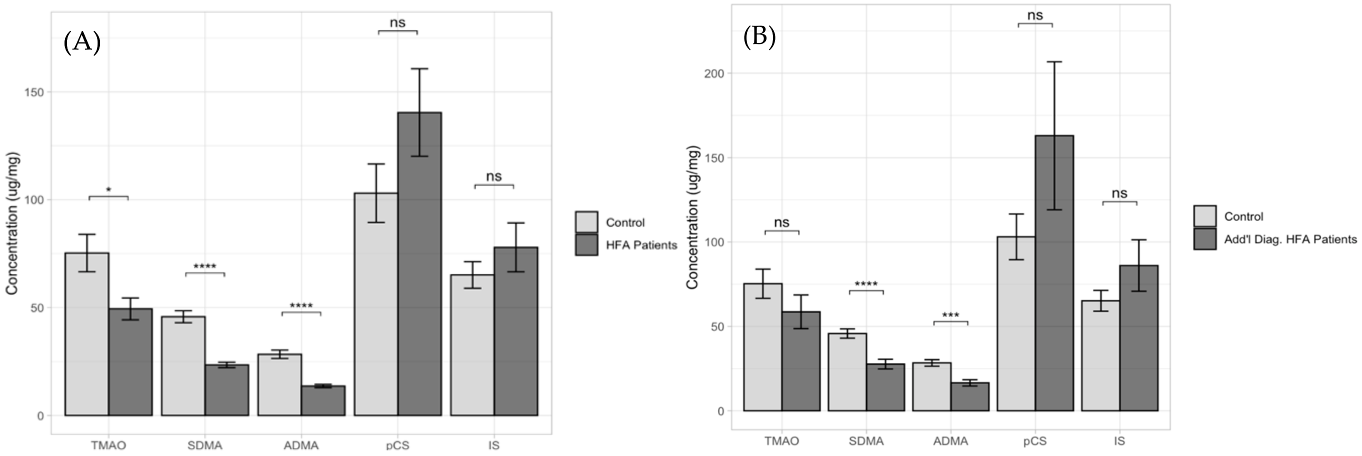

Figure S5. Levels of uremic toxins in the (A) HFA patients and controls; (B) HFA patients with additional diagnosis and control group.
